# Using YouTube Comments Data to Explore Postpartum Depression in Social Media: An Infodemiology Study

**DOI:** 10.3390/ijerph21111526

**Published:** 2024-11-18

**Authors:** Anila Virani, Bhupinder Nagra, Joyce O’Mahony, Juanita Bacsu, Jasjot Kaur Ghatore, Sourajita Panda

**Affiliations:** 1 School of Nursing, Thompson Rivers University, Kamloops, BC V2C 0C8, Canada; bnagra@tru.ca (B.N.); jomahony@tru.ca (J.O.); jbacsu@tru.ca (J.B.); 2 School of Nursing, University of Calgary, Calgary, AB T2N 1N4, Canada; jasjot.kaur1@ucalgary.ca; 3 Bob Gaglardi School of Business and Economics, Thompson Rivers University, Kamloops, BC V2C 0C8, Canada; spanda@tru.ca

**Keywords:** social media, postpartum depression, stigma, support systems

## Abstract

Background: Postpartum depression (PPD) is a prevalent mental health issue profoundly impacting both parents and their families. This study examines YouTube comments to identify common public discourse and prevalent themes surrounding PPD. Methods: We analyzed 4915 comments from 33 YouTube videos to provide a comprehensive picture of PPD-related discourse on social media. We analyzed data using engagement metrics and Braun and Clarke’s thematic analysis. Results: The engagement metrics indicated that public discourse is primarily focused on the stigma associated with PPD in men and celebrities, with related videos receiving significant attention and high engagement metrics score. Thematic analysis revealed two themes: (1) perspectives of stigmatized, stigmatizer and people in between; and (2) adaptation despite adversity. Conclusion: This study provides key insights into public discourse on PPD. It highlights the importance of family and community support and advocates for a healthcare system capable of addressing the needs of stigmatized populations. A significant finding of this study is the call for action to raise awareness and debunk myths about PPD. Misconceptions worsen stigma and deter help-seeking by affected individuals. Awareness initiatives are crucial to enhance public understanding of PPD symptoms, its impact on individuals and families, and the importance of parental mental health.

## 1. Introduction

Postpartum depression (PPD) is a group of symptoms that can negatively affect and alter the parent’s mood, attitude and behaviour. According to DSM-R-TR, it is marked by intense feelings of sadness and anxiety, as well as disturbances in sleep, energy and appetite [1]. Symptoms can start anytime during the pre- and postpartum period. The birth of a baby is a time for celebration as well as an overwhelming sense of responsibility as a family welcomes a new baby into their lives. It is also a vulnerable time for women due to postpartum physiological changes. The hormonal fluctuations, discomforts and lifestyle changes (sleepless nights and lack of social networking activities) make this transition challenging and pose a risk of developing mental health issues [2].

PPD is one of the most common mental health concerns globally. PPD is negatively associated with poor parental outcomes (irritability, poor sleep and appetite, social isolation, loss of interests, low self-esteem and thoughts of harming self or baby). It is also reflected in the negative behavioral, emotional and social development of infants and poor relationships with other family members [1,2]. According to a systematic review, 25.8% of women experience PPD, and infants born to these mothers exhibit 31% poorer outcomes compared to those born to mothers without PPD [3].

A well-known factor in minimizing the negative effect of depression is access to and provision of social support [4]. Perinatal women with inadequate social support are five times likely to experience PPD [5,6]. Social media has provided a common public ground to connect with others and express feelings with similar interests and issues. The freedom to be anonymous also provides an opportunity for parents to share their feelings openly and honestly. The increased use of social media to provide and receive support among parents is well documented. However, it can also lead to feelings of comparison, potentially exacerbating the symptoms of PPD [7,8]. The COVID-19 pandemic has intensified the challenges faced by new parents. During the pandemic, postpartum parents were particularly at risk due to the heightened stress and anxiety associated with COVID-19 pandemic-related restrictions such as social distancing. This led to a decrease in social support, thus negatively impacting overall parenteral mental health [9].

PPD social media research is primarily focused on developing predictive models to identify mothers at PPD risk [10], administering self-reported questionaries and surveys to predict the degree of depression [11] and online interventions for PPD [12,13]. For example, Zhang et al. [8] discovered a link between the content posted by new mothers on WeChat and PPD. They found certain types of posts, particularly those expressing negative emotions or challenges in motherhood, were associated with higher levels of PPD.

In past PPD research, most participants were women, able and motivated to participate in research [14,15]. These data lack the perspectives of those who do not wish to participate or do not have the time, energy or motivation to participate in research. Depression affects motivation and interest, and a high attrition rate (87%) is reported in a meta-analysis of online PPD interventions [12]. Moreover, many parents, although they intended to participate could not participate or did not show up due to situational changes [16]. Therefore, not only is social media and online forums an accessible source for parents to seek emotional and social support but also for researchers to capture these sometimes-underreported data. Recognizing the needs of new parents of diverse geographic contexts is also vital in the recovery of the physical and emotional well-being of parents after childbirth [6].

YouTube has 2.7 billion monthly active users, making it the second most popular social media platform globally. Users can post videos of varying lengths, utilize hashtags to target their audiences and interact through comments, likes and shares. Every minute, over five-hundred hours of content are uploaded, with videos receiving an average of seventy billion views. The platform’s user base is aged 18–34 with 54% of users identifying as men [17,18,19].

Researchers have identified social media as a powerful tool for reviewing public conversations about mental health, as it enables individuals to share personal stories, seek information about conditions and treatments, and exchange support with others facing similar challenges. By analyzing public discussions on social media, researchers can identify current trends in information. These trends can be related to specific conditions, particularly issues of discrimination and stigma, barriers to accessing mental health services and the unmet needs of these populations. Analysis of current discourses can also help in planning interventions accordingly [7,20,21,22]. To date, there are no studies that analyzed participants’ verbatim text publicly available on YouTube in relation to social support during the pandemic. Massell and colleagues [23] have investigated the progression of mental health trends on Twitter both prior to and during the pandemic. However, their study does not address PPD. The study commenced exploring the role of social media in offering and receiving social support for PPD to assess the impact of the pandemic on these social support dynamics. Initially, we deductively analyzed the data using the social support theory presented by Leahy-Warren [24], which describes several types of social support in relation to PPD. It was evident that YouTube was a major source of appraisal support. Sixty-nine percent of the comments were related to appraisal support and these findings are presented elsewhere [25]. However, a deep dive into the data revealed themes other than social support such as stigma. Therefore, the data were reanalyzed using a combination of inductive and deductive approaches using stigma frameworks such as the Mental Illness Stigma Framework (MISF) [26] and the Health, Stigma and Discrimination Framework (HSDF) [27]. Also, our initial purpose was to collect data during the pandemic to see how it affected social support. However, the word search of 7177 comments using COVID-19 pandemic-related words revealed only thirteen comments. Therefore, this paper aimed to analyze the comments of YouTube viewers to identify common public discourse and prevalent themes related to PPD.

## 2. Research Design and Methods

### 2.1. Ethical Consideration 

Ethical board approval was not required as we utilized the publicly available data on YouTube. To preserve the privacy of the commenters, we did not include their usernames. Any names used by the commenters in the comments were replaced with pseudonyms. However, the names of the people featured in the videos were kept the same. This approach ensured that the context was maintained while safeguarding the privacy of the commenters. Names and places mentioned in comments by viewers were replaced with pseudonyms, unless they were part of the video to provide context. Commenter quotes were modified by replacing words such as “moms” with “mothers” and “postpartum depression” with PPD to reduce the chances of retrievability.

**Figure 1:**
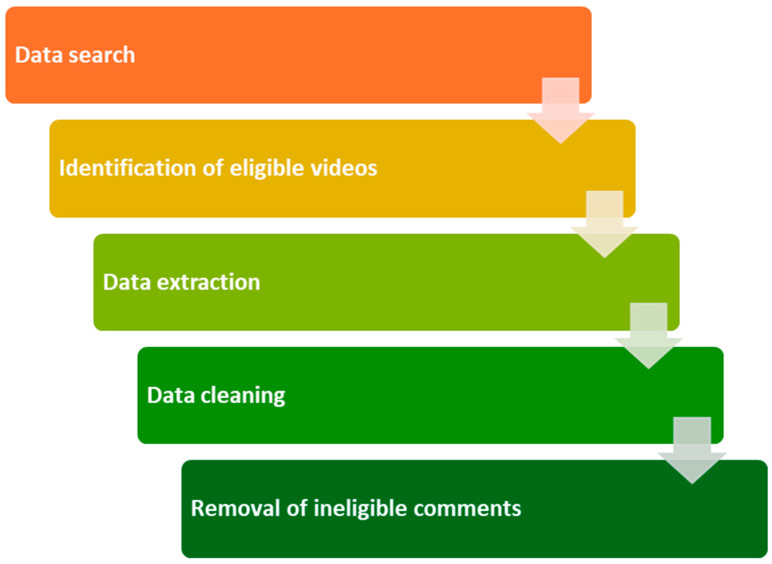
Methodological approach.

It is important to note that this effort cannot fully guarantee that the comments cannot be traced back to the commenters. However, researchers should diligently strive to protect people’s privacy beyond procedural ethics [28]. This section describes the methodological approach in detail as illustrated in Figure 1.

### 2.2. Data Search

We searched YouTube videos related to social support in PPD during the pandemic. We used the search terms Depression AND Postnatal OR Perinatal OR Postpartum OR PPD AND COVID OR pandemic using the Google advanced search filter. We retrieved the top ten videos for each month from January 2022 to June 2023 to capture the time when pandemic restrictions started to ease off to the WHO official announcement for the end of the pandemic. In total, we retrieved 180 videos.

### 2.3. Identification of Eligible Videos

We used the following ineligibility criteria to identify the relevant videos:

- No comments posted by the viewers: n = 52
- Non-English: n = 34
- Informational videos on PPD: n = 12
- PPD-related movies and advertisements: n = 3
- Sharing normal postpartum and parenting struggles: n = 4
- Neither focused on depression or COVID-19: n = 11
- Specific to mental health but not PPD or PPD was briefly mentioned: n = 30

**Figure 2:**
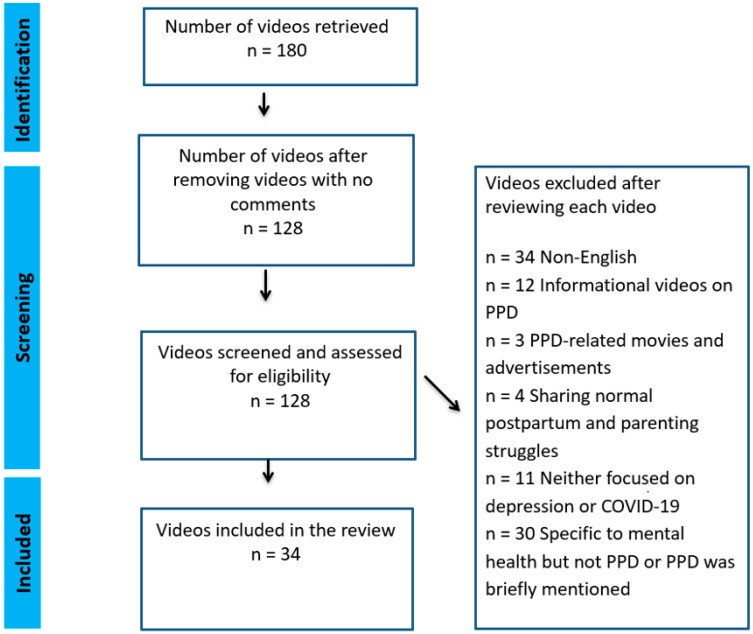
PRISMA flow diagram for the selection of eligible videos (Page et al. [29]).

Figure 2 shows the PRISMA flow diagram for the eligible video selection. We identified thirty-four eligible videos that focused on people’s firsthand experiences of PPD. These videos were created by affected parents and/or family members, or by newscasters, podcasters or YouTubers through interviews or by compiling publicly available news about affected individuals.

### 2.4. Data Extraction

We hired a Graduate Research Assistant (GRA) from the computer science department to scrap the data. The GRA used the scrapping code to populate the following data for each video in the Excel sheet for cleaning and analysis: (i) video name and URL; (ii) username; (iii) date and time when the comment was posted; (iv) comments; (v) reply count; and (vi) likes count. We retrieved a total of 12,717 comments including 8476 original comments and 4241 replies from thirty-four eligible videos.

### 2.5. Data Cleaning

The GRA cleaned the data using Python for the comments that were not in English (n = 76); duplicates (n = 166); comments consisting solely of emojis (n = 297); comments containing only HTML tags, URLs, random letters, entities and hashtags (n =134); comments with less than three words such as “thank you,” “beautiful”, “this is sad,” etc. (n = 626). We removed a total of 1299 comments during the cleaning process. We also removed replies to the original comments (n = 4241) as many of them were short and it was difficult to make sense of the replies without the context of the original comments. We also lost all comments from one video during cleaning. Therefore, in the final analysis included 7177 comments from thirty-three videos Participant’s quotes were cleaned for spelling and grammar to support readers’ comprehension of the results. Slang and inappropriate words were either removed or their spelling was replaced with asterisks.

### 2.6. Removal of Ineligible Comments

**Figure 3:**
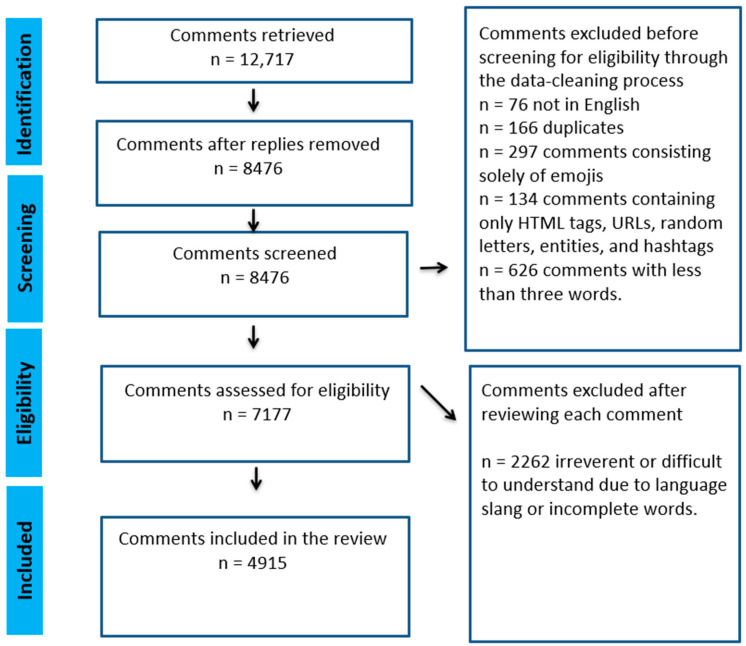
PRISMA flow diagram for the eligible comments (Page et al. [29]).

An undergraduate RA was hired to support the thematic analysis of the data. The URA identified 2262 as ineligible comments with the support of AV and JM. Comments were considered ineligible if they were irrelevant or difficult to understand due to language slang or incomplete words. After removing the ineligible comments, the remaining 4915 comments were used to analyze the data. Figure 3 depicts the PRISMA flow diagram for the eligible comments.

### 2.7. Data Analysis

We analyzed data by performing engagement metrics analysis of comments, replies, likes, views and hashtags of the thirty-three eligible videos, as well as qualitative thematic analysis of the comments (n = 4915) posted by the viewers using Braun and Clarke’s [30] thematic analysis.

Engagement metrics such as comments, replies, likes, views and hashtags provide valuable insights into users’ interactions with content. The number of comments indicates the level of viewers’ interaction with the content. A high number of comments suggest that the video has resonated with the audience and sparked discussions around the content. Similarly, a high reply count on comments reflects deeper interaction, as viewers are not only sharing their thoughts but also responding to others. This shows that the video has generated significant interest or debate, prompting viewers to engage in conversations. The metrics of likes and view counts are interrelated. Higher numbers of likes correlate with increased view counts, as “likes” create a positive association with the content and enhance the likelihood of the video being recommended to other users. Conversely, higher numbers of dislikes or a low number of likes tend to deter potential viewers. A hashtag is a word or phrase preceded by a # sign that categorizes content and increases its reachability to viewers. It allows users to explore related content by directing them to videos and channels associated with the hashtag [31,32,33,34]. 

The comments were analyzed using a combination of inductive and deductive approaches using Braun and Clarke’s [30] six steps of thematic analysis: (1) familiarization with the data; (2) developing initial codes; (3) searching for themes; (4) reviewing the themes; (5) defining and naming the themes; and (6) producing a report. The initial list of codes and themes was inductively developed by AV using a data-driven approach. AV and BN then revised the list over several meetings. We also used the MISF [26] and the HSDF [27] to guide our analysis. The final themes were reviewed by JM, a PPD expert and JB, a stigma expert. Any discrepancies were resolved through discussions. To ensure rigour, we maintained a detailed audit trail of our research process and reflexive records of emerging thematic maps on Miro and Canva as they were revised over time. In qualitative research, triangulating multiple methods or data sources is a strategy for testing validity by comparing and converging [35]. Our engagement metrics data supports our thematic analysis and provides a comprehensive picture of the prevalence of PPD stigma and strategies to mitigate stigma on YouTube.

## 3. Results

### 3.1. Engegment Mertics Analysis 

Engagement metrics analysis for the thirty-four eligible videos revealed that the following seven videos ranked high on one or more of these metrics. Table 1 details comments, replies, likes, views and hashtags for these videos.

- Erik has the daddy blues
- DanTDM opens on fame, postnatal depression and fatherhood
- Hayden Panettiere opens about struggles with alcoholism, postpartum depression
- Intervention: Severe postpartum depression sent Tiffany down a path to heroin
- Mom experiencing postpartum depression dies by suicide days after giving birth
- She thought they would not find out
- The Cordles on waiting till marriage, postpartum depression & getting hate online

**Figure 4:**
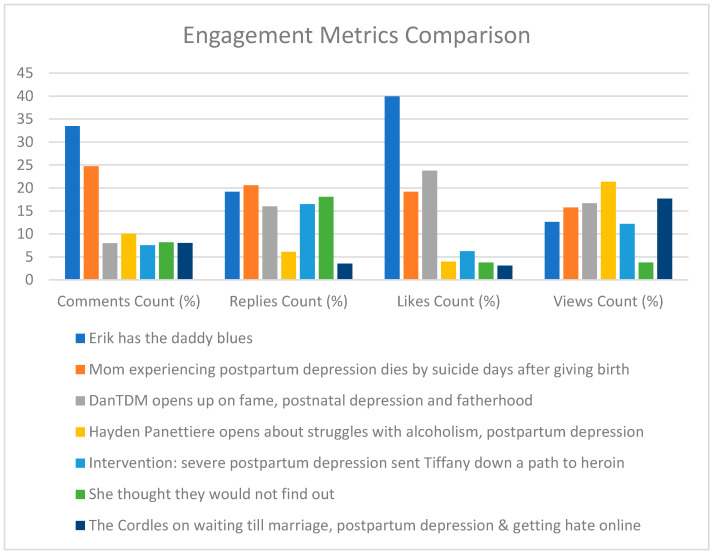
Comparison of engagement metrics among top videos.

Figure 4 compares engagement metrics such as comments, replies, likes and views across videos ranked in the top five for any of these metrics. This comparison identifies videos that resonate most with viewers in different engagement aspects. In both videos, “Erik has the daddy blues” and “DanTDM opens up on fame, postnatal depression, and fatherhood” high viewer engagement was shown, with comments discussion various aspects of PPD in men. The video, “Erik has the Daddy Blues” received the highest number of likes (n = 52,720) as well as the highest number of comments (n = 2426) comprising 29% of total comments. It also has the 2nd highest number of replies (n = 700) and ranks 5th for view counts (n = 470,430). The video “DanTDM opens up on fame, postnatal depression, and fatherhood” ranked top 3 on view count (n = 622,501), top 5 on reply count (n = 584) and top 2 on likes count (n = 313 39), though it had a small number of comments (n = 577). 

Of the eligible videos analyzed, 41% (n = 14), featured celebrities discussing their experiences with PPD. Celebrities included Hayden Panettiere, Mandira Bedi, Kylie Jenner, Maren Morris, Mena Suvari, Thomas Rhett, DanTDM and Natalie Grant. The video “Hayden Panettiere opens up about struggles with alcoholism, postpartum depression” received the highest view count (n = 797,004), 3rd comments count (n = 728) and 5th for the likes count (n = 5220). Another video that made it to the top 5 for the likes (n = 8260), and replies count (n = 601), was “Intervention: Severe postpartum depression sent Tiffany down a path to heroin” which also shared the subject matter of drug use in PPD. 

The video “Mom experiencing postpartum depression dies by suicide days after giving birth” and “She thought they wouldn’t find out” shared the debilitating effects of PPD on the family. The video “Mom experiencing postpartum depression dies by suicide days after giving birth” shared a mother’s story of committing suicide due to PPD. In contrast, the video “She thought they wouldn’t find out” shared the story of the mother who killed her child while suffering from PPD. “Mom experiencing postpartum depression dies by suicide days after giving birth” has the highest reply count (n = 751) and has the second highest number of comments (n = 1792). It has the 3rd highest likes count (n = 25,312) as well as the 4th highest view count (n = 587,644). “The Cordles on waiting till marriage, postpartum depression & getting hate online” ranked 2nd for view count (n = 658,891) and 5th in comment count (n = 581). 

The hashtags revealed mixed results that show the complex mechanism of hashtag features. The total number of hashtags was sixty-three with a maximum of six hashtags per video. For example, the video “Hayden Panettiere opens up about struggles with alcoholism, postpartum depression” has six hashtags and ranks within top five for comments., views and likes count, showing the importance of hashtags in reaching to wide audience. However, a video titled, “Doctor suffered from postnatal depression! One born every minute” also has six hashtags but did not make it to the top five videos depicting another complex factor that it is not only important to have a good number of hashtags but also ones trending and popular among users. In contrast, the video “Erik has the daddy blues” has no hashtag and yet has the highest comment, likes count and ranks within the top five videos for replies and views reflecting that a video can still have a bigger reach despite no hashtag. Hashtag #postpartumdepression is associated with 5800 videos and 3000 channels on YouTube yet only one video “#mandirabedi postpartum depression story: struggles, advice for others” out of thirty-four eligible videos had the hashtag of postpartum depression and did not make it to the top five videos in any category. Table 2 provides the list of hashtags and their frequencies in eligible videos.

### 3.2. Thematic Analysis 

Our qualitative data indicated two themes: (1) perspectives of stigmatized, stigmatizer and people in between; and (2) adaptation despite adversity.

#### 3.2.1. Theme 1: Perspectives of Stigmatized, Stigmatizer and People in Between 

Perspectives of stigmatized, stigmatizer and people in between revealed three subthemes: (1.1) myths and realities; (1.2) shared experiences of fear and anxiety; and (1.3) family’s experiences of PPD.

##### Subtheme 1.1: Myths and Realities

The following four myths were dominant in our data:

- Celebrities are too privileged to have PPD
- Men cannot have PPD
- PPD is not real (it is a made-up disease)
- Parenthood is full of joy.

Celebrities were mostly trolled on the YouTube platform. Videos that captured Hyden Panettiere, an American actor’s struggle with PPD reflected, “It is extremely hard to feel sorry for her. The woman has possessed untold levels of privilege from an early age. In a relationship with a man like WK from age 20… and a multi-millionaire in her own right at an incredibly early age. Most of us cannot even fathom that. I suspect she is very spoilt and has no idea what it is to be truly alone. The only thing I felt sorry for her about is being so short!” Some viewers expressed celebrities may experience PPD, but their access to money and resources makes it less severe as com-pared to the poor or average-income people. “Good for her but I am a little tired of these celebrities coming out with stories of addiction, mental health and so forth type stories of redemption/surviving the struggle when they have more resources than everyday 9–5 workers who must deal with this and while struggling to keep food, rent, normal monthly bills paid. Congratulations you have been able to use your millions to make your struggle less debilitating, who cares.” Some people expressed that celebrities fake these illnesses and struggle to get fame. “Publicity for her [Hyden Panettiere] new film. Do not necessarily blame her because she is in show business and that is what people do, but commenters should not read too much into it. She is an actor and knows what she is doing”.

While most people trolled these celebrities, others contributed to the discussion by ad-dressing the stigma. “I have no blame on her [Hyden Panettiere]. I am not a celebrity, but I [suffer from] depression in my life and have also been separated from my daughter which I never could ever believe, it might happen to me. Never try to step into someone’s shoes or blame or judge. We never know the reasons behind it.” 

Another common myth was about men experiencing PPD. Some commenters expressed skepticism, “I am sure someone has clarified but cis men cannot get PPD because PPD or PA are caused by hormones from pregnancy.”, “My feminism is screaming. Man wants to be special.” and “PPD is for women lol [Laugh out Loud].” Some commenters transformed the discussion by de-bunking the myth. For example, “PPD and anxiety are well-documented for fathers. and women who have PPD have a valid cause and effect for being depressed. We tend to validate women’s mental health more than men’s”.

People also shared the myth that PPD is a made-up disease. “We really need to understand the devil does these things like depression, anxiety and any other thing like that Lean on the Lord he is ever strong much stronger than we are way much!” Some myth-busters responded to such comments by emphasizing that “PPD is not a choice.” and “The thing with depression and its variations is that for so long it was something seen as a choice, as one being weak, and my favorite one: a female thing. not only depression but PMS, and PPD. These things are real and extremely dangerous”.

Another common misconception is that parents should always feel joyful and should not express feelings of sadness. If sadness does occur, it is believed to be brief and temporary, at-tributed solely to hormonal changes. Otherwise, it is seen as a longing for attention and the desire for the life parents had before the baby was born. “I am sick of postpartum excuses. She could have found support. A baby is a joyous event, baby blues do happen, happened to me for a few days, partly because of the attention when you are pregnant, then the fear, oh this is a big responsibility, but most get over then”.

In response to such comments, some comments mentioned the normal struggle of parenthood and highlighted the stress that comes with complicated pregnancies, delivery, and post-birth trauma. “This is a real and predictable problem that takes a person and dramatically crashes their hormone balance, at the same time, do not let the new mother sleep soundly for six months. Sleep deprivation for only 3 days can cause hallucinations in many people. Then make the body produce milk for the baby and add perineal pain and sore breasts.” and “So many women are ashamed to admit to feeling down because having a baby is supposed to be such a happy time in your life and for the most part it is, but it also a major life change and hormones are not a joke! Depression after baby does not mean it was not amazing and does not mean you are a bad mother”.

##### Subtheme 1.2: Shared Experiences of Fear and Anxiety 

Fear and anxiety were a prominent sub-theme among parents grappling with PPD, and people contemplating becoming parents or having more children. Fear was intertwined with sensations of losing control, facing obstacles in accessing care during the pandemic, fear of social isolation and contemplating suicide.

Fear was exemplified by comments such as “It was such a long, ugly, dark, lonely, broken empty road.”, “This is an unbelievably sad and horrific period.” and I was “Afraid of the stigma”. Comments on the risks and attempts of suicide due to PPD and other mental health conditions were also shared. “I ended up in the emergency twice as I was actively suicidal and could not stop thinking about ways to kill myself.” and “Most mothers with PPD who commit suicide, do so between 9-12 months postpartum which coincides with when I truly hit rock bottom.” These remarks reflect a deep seated hopelessness and fear associated with PPD, highlighting the pro-found sense of dread and despair it encompasses, along with the apprehension of suicidal ideation. 

Fear also manifested in relation to inadequate access to care. One individual stated, “There are those who have not come forward either to the GP or who suffered in silence, add this to the pandemic lockdowns.” Many parents felt overwhelmed with fear of judgment and dismissal. One commenter admitted, “The anxiety about getting help/getting medication gives me such anxiety as well so I have not been able to get the help I truly need.” Isolation, both physical and emotional, amplified the fear and anxiety and was exacerbated by the pandemic. Lockdowns and restrictions intensified the feelings of loneliness and separation from support networks. “After my first child, I had PPD. I would burst into tears suddenly. I felt so completely alone. It was like I was the only one by myself. I had no friends. No one to talk to.” and “I got pregnant right before the pandemic… I felt so isolated, and it really affected my mindset”.

Some commenters questioned and contemplated their decision to have children. “This is why I refuse to become pregnant. I do not want to put myself in a possible depression episode. I do not want my mental health to decline for a baby. I know having kids can be exciting, but they are not worth it if you are going to end up severely depressed or even suicidal.” Individuals with prior mental health issues also shared their anxiety. “I wonder if it is more common for people who have already had mental health issues. I have had major depressive disorder for over 15 years now. I am too scared to have a child”.

The discussion also revolved around parents who were planning to have more children. Commenters urged caution, “You need to decide if it is worth the risk.” and “I hope women with PPD, and their partners think long and hard before having more children after one episode.” Another contributor emphasized the gravity of this decision stating, “I have to say that if it were me, I would never have even attempted to get pregnant again. Knowing how bad the first time was. It should have been foreseen by the doctors that it would be worse with twins” and “I hope future parents take this seriously. Is your partner’s potential death worth having a child even with the best laid out plan? If it is, then go for it. If it is not, you do not have kids”.

##### Subtheme 1.3: Family’s Experiences of PPD

Children who had mothers with PPD shared distressing examples of the profound physical and emotional impact. “She was only sixteen and threw me against a wall. She was hospitalized for almost seven months and admitted years later. She never bonded with me or felt maternal instinct.” and “My own mother told me when she had my older brother, she did not even believe he was her baby, my old boss told me once she had her son, her PPD was so terrible that she had to fight the urge to throw her baby off a bridge.” These testimonies shared the devastating emotional consequences PPD has on trust, emotional safety, and overall well-being within familial relationships. 

The effect of PPD on the family unit reshaped roles and responsibilities. Family members assumed the role of primary caregivers for the infant and navigating the complexities of childcare while grappling with the emotional toll of their loved ones struggling with PPD. “My sister had PPD. We did not know until recently, but we raised my niece.” and “Thank God we have supportive grandparents. My mother gave my brother and me to my grandparents to raise for one year while she recovered. We have had a normal family relationship since then, I am off to college and my younger brother is off to high school. She loves us both now and would do anything to protect us.” This narrative emphasized the importance of familial support in fostering recovery and positively shaping the lives after such tragedies. 

#### 3.2.2. Theme 2: Adaptation Despite Adversity

People showed adaptation to PPD despite recognizing its adversity and the challenges it brings to a person’s physical, mental and social health. Three subthemes emerged: (2.1) lack or availability of support systems; (2.2) bounced back or still there? and (2.3) advocacy and awareness.

##### Subtheme 2.1: Lack or Availability of Support Systems

Comments indicated both, the presence and absence of support systems. Commenters discussed the role of partners, family, community, society, healthcare system and availability of multifaceted treatment approaches in exacerbating or easing the effects of PPD.

The role of a partner was found to be pivotal in either worsening or alleviating the effects of PPD. Some participants expressed concerns regarding the insufficient support from partners postpartum, “I think 90% of postpartum causes are husbands. They think it is not a big deal to keep a baby inside your belly for nine months and then give birth and start taking care of him/her, staying awake at nights and not able to sleep in the day too. A woman goes crazy due to lack of sleep and hearing this heartbreaking stuff from husbands”, and “many men chose not to acknowledge the effects on a woman’s body and mental health due to having children”.

Conversely, some comments reflected positively on partners, crediting them in helping with PPD “It is so cool how supportive your husbands are”, and “It got so bad that my husband had to calm me down multiple times from episodes of me screaming that I wanted to die”. Amidst the challenges of child birthing during the COVID-19 pandemic, the presence of a supportive partner was found to have a great significance, “I was twenty when I had my first baby, in the height of COVID. Had a c-section and only my husband was allowed in the hospital the entirety of our hospital stay”. Additionally, there were calls for parental leave for fathers, indicating the significance of equitable distribution of caregiver responsibilities in promoting maternal well-being and family cohesion. “A mother should never be the only one taking care of her kid. Fathers should be allowed to stay home for at least six months during the first three years of the child’s life”.

The importance of family emerged as crucial in addressing PPD. The significance of support systems was reflected in statements such as “If she ever felt different or if I noticed her not being herself, we would talk and seek help. One night about three weeks after the most beautiful little girl in the world was born, she came to me and said, mother I need a break. I took the baby and let her rest, no problem. She just told me a couple of months ago. Mother I was going to hurt my baby that night I brought her to you”.

In addition to the family, community support in aiding new parents facing PPD became evident. There was a connection drawn between community involvement and the prevention of harm to children. “This is a prime example as to why everyone should step up to the plate and volunteer to help with mothers in your community that may be going through a similar crisis. Sometimes just offering to watch the children for an hour or two can relieve a lot of stress for a mother. Itis a shame that that poor baby had to suffer because the mother did not feel like she had any other recourse and did not seek any help” and “If you have someone in your family or social circle who just had a child, check up on them to make sure they are coping healthily. All new mothers need help sometimes, but some do not ask for help because they have been conditioned to deal with everything themselves”.

PPD sufferers felt stigmatized to receive support from the healthcare system. Comments identified obstacles that parents encounter in both acknowledging their condition and seeking appropriate treatment. “They always say women are lying about postpartum so there you go, hope you’re happy now little children do not have a mother”, and “Even in 2010 doctors here in [name of the country] refused to understand my feelings and said you are just not willing to accept the joys of motherhood”. Another individual shared financial struggles, “My insurance could not pay for me to have therapy with providers that could take new patients. and those that did take my insurance were not taking new patients when I was going through my PPD”. People advocated for a more attentive and ongoing approach from the healthcare system to prevent the harmful effects of PPD. “More could be done to prevent events like this from happening. How long does it take for a nurse or doctor to make a two and a half minute phone call to all the new mothers, like once a week or so to check in on them for the first six months? Often times people are too ashamed or embarrassed to ask for help, or scared even, at the thought of their babies being removed from their care, but if help is offered and process and procedures are explained, it is a different story”.

Lastly, some voices advocated for the adoption of multifaceted treatment and holistic approaches to address PPD. Commenters shared “functional/integrative healthcare can help”. People suggested “meditation”, “mindfulness”, “counselling”, “cognitive behavioural therapy”, “talk therapy”, “natural supplements” and “exercises” to cope with PD symptoms.

##### Subtheme 2.2: Bounced Back or Still There?

Findings revealed a nuanced narrative surrounding PPD, capturing both the negative impact and the resilient spirit that persists amidst adversity. Discussions within videos shed light on PPD exacerbation by the compounding effects of childbirth trauma and the overshadowing presence of COVID-19 pandemic. “I am still struggling with PPD three years on, and I have also been left permanently damaged from labor. Nobody cared because COVID took over. PPD is awful”.

The interplay between PPD’s daily existence imposed enduring consequences on parental wellbeing. “PPD is no joke. Had my daughter at 23 weeks and she is now 4 years old and I still have my moments/weeks or months where it is PPD all over again. I know it is not; they would consider it depression now and some anxiety but regardless, I feel like what I did when I had PPD. It is truly crippling and debilitating. PPD is not talked about enough and there is not much education out there about it, so it creates this negative stigma in society”, and “After my second pregnancy I am battling severe PPD, and my challenges have put me in a dark hole. I am still fighting and if I continue to reach for help, I can get better”. These firsthand accounts revealed the recurrent episodes, underscoring the persistent nature of the condition. It also highlighted the impact of PPD on daily life and mental wellbeing, long after childbirth and the ongoing societal stigma surrounding this issue. 

Despite the horrific ordeal of PPD, narratives emphasized the transformative power of treatment, offering a beacon of hope amidst darkness and foreshadowing resilience. “The day came when I did not want to live like this and took an overdose, they saved me. I did not get better till Susan was five, but I have been on antidepressants since. She is twenty-four now. Please get help if you feel like I did, it is just an awful condition”, and “ So much past traumas had triggered me like never before and I felt crazy. I took medication and did therapy. It helped me tremendously, but it was horrific”.

Religion emerged as a source of strength for many commenters, providing support for bouncing back and providing a sense of purpose for people coping with PPD. One commenter expressed the role of faith in overcoming adversity and finding comfort in the turmoil of PPD, “God has helped me have victory over the course of my life in different areas that I have struggled with however I have not made it through to the other side. What I realize is that I will never live without no fear. However, I can learn to walk through it with the father and I know he is with me and for me always. however, amid the trial we can speak out and help others”.

##### Subtheme 2.3: Advocacy and Awareness

PPD-related myths and comments from the stigmatizers were common among YouTube viewers. However, some people transformed these discussions to create social media, a place for raising awareness and advocating for the stigmatized populations.

A common suggestion was more awareness of PPD for parents and the public. An individual stated, “What a wonderful documentary. This should be shown to pregnant mothers and their families. we should have an awareness and be prepared to hopefully prevent this, not only for the parents but for the kiddos. Healthy kids and families are in everyone’s best interest”, and “Thank you for sharing your stories! it is very educational. Also. I will be able to make sure my friends and family know about this because it’s important and it could save lives”. One commentator beautifully shared the importance of awareness at all levels and advocated for support for new mothers.

One commentator beautifully shared the importance of awareness at all levels and advocated for support for new mothers.

“Bringing awareness to mothers experiencing PPD is of utmost importance in our society. It is a topic that requires compassion, understanding, and support from all. PPD is a real and serious mental health condition that affects countless mothers around the world, and yet it often goes undiagnosed and unaddressed. By raising awareness about PPD, we can help break the stigma surrounding it and create an environment where mothers feel comfortable seeking help and support. It is crucial to educate not only mothers but also their partners, families, healthcare professionals, and the public about the signs, symptoms, and impact of PPD. We must emphasize that PPD is not a reflection of a mother’s character or ability to care for her child, it is a biological and psychological condition that can be triggered by a com-bination of hormonal changes, sleep deprivation, and the overwhelming demands of motherhood. By understanding this, we can provide empathy and encouragement to mothers who are experiencing this challenging journey. Bringing awareness also means advocating for better mental health resources and support systems for new mothers. This includes promoting accessible and affordable mental healthcare services, creating safe spaces for mothers to share their experiences without judgment, and implementing comprehensive screening programs during and after pregnancy. By amplifying the voices of mothers who have faced or are currently facing PPD, we can create a network of support and understanding. It is essential to provide platforms for open discussions, where women can share their stories, offer advice, and connect with others who have been through similar experiences. Together, we can let these mothers know that they are not alone, and that help is available. Lastly, it is vital to remember that PPD can have long-lasting effects on both the mother and her child. By raising awareness and providing support, we can contribute to the well-being and healthy development of families. Let us strive to create a society where every mother feels heard, validated, and empowered as she navigates through the challenges of motherhood”.

## 4. Discussion

We conducted the engagement metric analysis of thirty-three YouTube videos. These metrics included the number of comments, replies, likes, views, and hashtags. The growing popularity of social media allows users to engage in activities such as creating profiles, interacting with others, expressing opinions and emotions, and sharing firsthand experiences. When users engage with posts through comments, replies, likes, and views, algorithms interpret this as valuable content, increasing its visibility. These metrics reflected the video’s popularity and high audience engagement, offering real-time data that captures current public perceptions of PPD and allows for monitoring changes over time [31,34].

The engagement metrics data indicated the discourse of PPD on YouTube towards topics such as the stigma of PPD in men and celebrities, and the debilitating effects of PPD on individuals and their families. While hashtags help categorize content and facilitate joining specific conversations [32], our study showed mixed results for hashtag use. Interestingly, hashtags specific to PPD were not commonly used in the popular videos that scored high on engagement metrics. 

The presence of celebrity figures in videos about PPD appears to play a role in shaping public awareness. The high engagement metrics associated with videos featuring celebrities, such as Hayden Panettiere and Kylie Jenner, suggested that celebrity involvement may increase visibility and relatability, potentially normalizing discussions around PPD. Such narratives might encourage viewers to engage more actively, perhaps as they see familiar public figures openly discussing mental health challenges.

Moreover, videos that delve into severe PPD outcomes, including substance use and suicide, generated substantial viewer engagement, as evidenced by high comments and replies count. Likely, resonating with viewers on a deep emotional level, prompting both reflection and dialogue. This content highlighted the serious implications of untreated PPD, and captured the viewers’ attention more effectively, underscoring the public’s interest in understanding the full spectrum of PPD’s impact on individuals.

The qualitative findings highlighted perspectives of stigmatizers through myths and discouraging comments, and views of the stigmatized, such as fear, anxiety, and family role disruptions. Personal and family testimonies reflected the adoption of PPD despite its adversity. The presence or absence of partner, family, and community support significantly affected coping mechanisms. Some individuals managed to bounce back, while others continued to struggle with the crippling effects of PPD. Suggestions to raise awareness and advocacy were also shared to help PPD sufferers.

Our qualitative findings can be explained by the Mental Illness Stigma Framework-MISF [26] and the Health Stigma and Discrimination Framework-HSDF [27]. The MISF [26] provides perspectives of both stigmatizers and the stigmatized, evident in our study through comments that reflected stereotypes, prejudice, and discrimination. The MISF outcomes of stigma include di-minished self-esteem, increased psychological distress, reduced quality of life, social exclusion, and reduced help-seeking behaviours which were also shared by the commenters in our study. This framework aids in identifying these impacts to inform interventions aimed at reducing stigma and improving the well-being of individuals with mental illness.

The HSDF [27] offers a comprehensive approach to understanding and addressing health related stigmas. The framework identifies key domains in the stigmatization process: drivers and facilitators, stigma marking, and stigma manifestations. These domains collectively shape out-comes for affected populations, organizations, and institutions, influencing overall health and society.

Beliefs such as, PPD is not real and that fathers experiencing it are incompetent, drive stigma. A study on Reddit posts highlighted that men experiencing PPD often come to healthcare professionals’ attention later due to limited awareness of symptoms and reluctance to seek help [36]. The stigma of PPD can lead to depression, anxiety, and decreased quality of life. PPD in men poses a strain on family relationships and can potentially lead to possible emotional and behavioural issues in children [37]. Facilitators such as cultural, social and gender norms were expressed in the forms of PPD-related myths such as men cannot have PPD, or parenthood is full of joy. Drivers and facilitators influence stigma marking based on health conditions such as PPD or perceived differences like race and gender, manifesting in various stigma experiences and practices, including stigmatizing behaviours and discriminatory attitudes [27]. 

Storytelling is a powerful method used in public health campaigns to make the experiences of individuals with diseases more relatable. Celebrities sharing their mental health issues on social media can make people less likely to judge others or feel ashamed about having mental health problems. Seeing celebrities discuss their mental health can make these issues seem more normal and reduce negative stereotypes and stigma [38]. A study of 157 celebrity health stories found that these narratives, including those about mental health, help reduce stigma and normalize health issues by increasing public knowledge and encouraging self-disclosure [39]. In our study, most people expressed stigmatizing behaviours and discriminatory attitudes towards men and celebrities for sharing their struggles. However, some people provided supporting and encouraging comments that conveyed compassion and inspiration towards these fathers and celebrities.

Stigma’s pervasive impacts on mental health are particularly pronounced in PPD, affecting parents’ access to and acceptance of necessary healthcare services. The findings of this study, particularly the theme of “adaptation despite adversity,” highlighted how stigma influences resilience and advocacy while simultaneously inhibiting healthcare access and the acceptability of treatment. This insight emphasized the necessity for comprehensive PPD interventions that address stigma, a critical barrier to effective care. Lack of support from healthcare providers was one of the most significant findings that call for a more robust healthcare system attentive to the needs of parents suffering from PPD. Training healthcare providers to conduct comprehensive assessments and regular check- ins with new or at risk parents is crucial, Educating partners and families further enhances support networks, facilitating better access to treatment and services. Reducing stigma and increasing awareness can improve help-seeking behaviors among parents. Stigma negatively influences attitudes toward seeking professional psychological help, while mental health literacy positively affects these attitudes highlighting the need for educational interventions. Involving partners and families in the educational process can further mitigate stigma, creating a more supportive environment that promotes treatment adherence and reduces feelings of isolation among parents experiencing PPD [40,41]. 

Our study also indicated that social media serves as a place for people to share their mental health symptoms and experiences. McLellan et al. [42] suggested sharing experiences online can help reduce isolation, offer hope, increase confidence, allow learning from others, and decrease stigma. Watching videos about stigmatizing beliefs and experiences can validate the experiences of those with mental illness as evidenced by testimonies mentioned in our study. Sharing firsthand experiences of stigmatization may have similar benefits for the commenters. Future studies can focus on exploring these benefits for people affected by PPD.

Social media also serves as a great platform for peer support networks such as online support groups offering women dealing with PPD an avenue to connect with others, gain information, and find encouragement and hope [20]. Naslund et al. [7] reported young individuals suffering from moderate to severe depression tend to prefer social media communication over in-person. Our study is an example of people using YouTube comments to provide each other with appraisal support. The viewers’ comments on the video “The craziest decision Natalie Grant’s ever made.” is an example of such support where people appreciated her story, shared their struggle, and used religion as a source of support in times of darkness. Most commenters shared kind messages for Nattalie and others who shared their stories. 

Addressing PPD-related stigma requires a multifaceted approach. Public education campaigns can change societal attitudes, dispel myths and reduce prejudice. Support networks and peer support groups can also combat stigma, providing validation and practical advice. Social net-working sites can raise social awareness among the public and within one’s family and friends. Events and trends can help make family members aware of PPD symptoms and what to watch for in new patients [43,44]. By addressing stigma, we can improve the lives of those affected by mental health conditions and create a more understanding and supportive society.

## 5. Future Prospects

Future studies should delve into the role of hashtags, algorithms, and user interactions in shaping social media discussions on PPD and related mental health content. Investigating these elements can provide an understanding of how platform structures impact the accessibility and distribution of mental health discourses [45,46]. Conscious use of hashtags can also direct traffic to content that reduces stigma and supports stigmatized individuals, fostering resilience and reducing isolation. 

This study highlighted several directions for enhancing PPD care. Prospective actions should focus on stigma reduction by creating a healthcare system sensitive to the needs of PPD sufferers, improving public awareness, developing supportive policies and strengthening stigma reduction research. Future interventions should integrate stigma-reduction modules into healthcare and patient education, creating a more accepting healthcare environment for those affected. Enhanced provider training, including thorough assessments and routine check-ins, is crucial for better support, enabling healthcare providers to respond sensitively to PPD cases. The integration of standardized screening tools could further streamline provider efforts to detect and monitor PPD, allowing for timely and effective intervention. For instance, tools like the Edinburgh Postnatal Depression Scale and the Beck Depression Inventory can support healthcare providers in identifying symptoms early and personalizing treatment pathways [47,48]. Moreover, incorporating culturally sensitive care practices ensures that interventions are respectful and effective across diverse populations, further enhancing the overall support system for those affected by PPD [47]. Systemic issues, for instance, provider shortages and treatment limitations, along with patient challenges such as stigma, time constraints, and physical access difficulties, hinder PPD treatment. Implementing virtual care and collaborative care models can help overcome these barriers and improve access to effective treatments [2]. Addressing the wider economic and societal costs of untreated PPD reinforces the need for a sustainable and equitable mental healthcare system that is welcoming to the people and families experiencing such conditions. 

There is an urgent need to raise awareness and correct misconceptions about PPD among the public. PPD-related myths contribute to stigma and discourage affected individuals from seeking help [49]. Public awareness campaigns are vital for improving the knowledge of PPD symptoms, the devastating effects that PPD can have on individuals and families and the importance of supporting parents. The consequences of PPD can be severe such as chronic illness, disability, suicide, and infanticide. The impact extends beyond individuals to families, communities, and society, with significant economic burdens on the healthcare system. Policy-level support for a comprehensive PPD care framework, including counselling, therapy, medication, and community programs is an essential step for long-term mental health outcomes Policies supporting the integration of standardized screening into routine postnatal care could facilitate earlier intervention, reduce long-term healthcare burdens, and provide consistent support for families navigating PPD [48,50].

Future stigma reduction research should be directed toward developing tailored frameworks and creating new evaluation tools that support culturally sensitive care. Involving policymakers, providers, patients, and other stakeholders in the research process can boost engagement and support intervention implementation. Increasing stigma research capacity is also crucial. Policymakers play a significant role in shaping supportive policies and securing funding. Creating opportunities for training and funding for stigma researchers is vital to advancing this field [47,51,52].

### Limitations

This study has several limitations that may impact the generalizability of its findings. First, with only thirty-three YouTube videos analyzed, the sample may not fully capture the broader discourse on PPD across social media platforms or with varied cultural contexts. Additionally, the study’s reliance on YouTube engagement metrics introduces possible selection bias, as content visibility is influenced by platform algorithms and user demographics. This may potentially skew results towards highly engaging content rather than providing a balanced representation of PPD experiences. Furthermore, our analysis primarily focused on visible comments, likes, and views, which may overlook private or less visible support for individuals affected by PPD. Lastly, the study was limited to English language content, likely excluding perspectives from non-English speaking users, which could restrict the scope of cultural insights into PPD stigma.

## 6. Conclusions

This study aimed to examine the common public discourse and prevalent themes sur-rounding PPD through the analysis of YouTube viewers’ comments. The study revealed significant insights into the stigma associated with PPD, highlighting the strong presence of societal mis-conceptions and negative attitudes on social media. Engagement metrics showed that the struggles of men and celebrities with PPD received considerable attention. This suggests that social media has significant potential as a platform for advocacy and support, bringing crucial issues to the forefront and fostering public discourse. The qualitative data yielded important insights into the perspectives of stigmatizers and stigmatized and individual’s efforts to advocate for stigmatized and raise awareness. Viewers’ narratives emphasized the critical need for community and healthcare provider support in identifying parents at risk for PPD and providing timely assistance. Healthcare providers should incorporate comprehensive and timely assessments, periodic check-ins, resource sharing and educating partners. The early identification of PPD and seeking timely support is critically necessary. The birth of a baby, while a joyful event, also brings overwhelming responsibilities that can negatively impact parents’ mental health. This research underscores the necessity for further efforts to improve mental health outcomes for parents. In-creasing awareness, reducing stigma, debunking myths, and creating supportive policies are essential steps toward transforming the discourse around PPD. The study highlighted the dynamic nature of stigma and its impact on behaviour and daily life. The persistence of stigma, despite the attention brought by celebrities and men sharing their experiences, signifies the need for continuous efforts in advocacy and education. Social media can serve as a powerful tool in this regard, providing a platform for sharing personal stories and fostering a sense of community among those affected by PPD.

## Figures and Tables

**Table 1:** Comments, replies, likes and views count for top videos.

Video Name	Comments Count	Reply Count	Likes Count	View Count	Hashtags
Erik has the daddy blues	2426	700	52,720	470,430	No Hashtags
DanTDM opens up on fame, postnatal depression and fatherhood	577	584	31,339	622,501	#LADbible #UNILAD
Hayden Panettiere opens about struggles with alcoholism, postpartum depression	728	223	5220	797,004	#GMA #HaydenPanettiere #Scream #Scream6 #alcoholism #postpartum
Intervention: severe postpartum depression sent Tiffany down a path to heroin	548	601	8260	455,309	#Intervention
Mom experiencing postpartum depression dies by suicide days after giving birth	1792	751	25,312	587,644	#postpartum #suicideprevention #gma
She thought they would not find out	593	660	4960	140,442	#documentary #psychology
The Cordles on waiting till marriage, postpartum depression & getting hate online	581	129	4105	658,991	#unplannedpodcast #mattandabby #thecordlefamily

**Table 2:** List of hashtags and their frequencies in eligible videos.

Hashtags Category	Hashtags
PPD	#postpartumdepression
Mental health	#mentalhealth (n = 5)
	#suicideprevention
	#depression
	#alcoholism
Parenting	#postpartum (n = 5)
	#Motherhood
	#birthvlog
	#laboranddelivery
	#mybirthstory
	#oneborneveryminute
	#oneborn
	#childbirth
	#Parenting
General hashtags	#documentary (n = 2)
	#people
	#WhatsUnderneath
	#Intervention
	#psychology
Brand or individual/family hashtags	#gma (n = 7)
	#thecordlefamily
	#GravitasFilms
	#HDFilms
	#RealFamilies
	#ENews
	#UNILAD
	#LADbible
	#unplannedpodcast
Celebrity-related hashtags	#HaydenPanettiere (n = 5)
	#kyliejenner (n = 2)
	#kardashians
	#scream
	#nashville
	#Scream
	#Scream6
	#mandirabedi
	#Heroes
	#Celebs
	#Actor
	#celebrity
	#MarenMorris
	#mattandabby
	#TheRundown

## Data Availability

The data are publicly available on YouTube.
